# Institutional Housekeeping under the Poor-Law

**Published:** 1918-01-12

**Authors:** 


### HOW NOT TO RUN A WAR HOSPITAL.

### Institutional Housekeeping Under the Poor Law.

THE Lewisham Board of Guardians has obtained from Mr. W. R. Owen, the deputy-clerk, a report, part of which is epitomised below, upon such points as the duties of the steward and his staff, and the method of receiving and issuing stores. The system of ordering goods, therein dealt with, is one common in principle to all Poor-Law institutions, and is to a large extent governed by the Orders of the Local Government Board. The report is of great interest, for it displays with considerable candour the general want of system, amounting almost to *naïveté*, which it is to be feared characterises the majority of Poor-Law institutions owing to the fact that they are for the most part governed by amateurs in the art of catering and the control of supplies. As it stands it is a most valuable commentary on the fact that Poor-Law catering costs double and sometimes treble per head as compared with the catering in such hospitals as Guy's and St. Thomas's.

### AN UNSATISFACTORY METHOD.

Briefly, the system of ordering consists of requisitions for the estimated quantity of goods required during each week. These requisitions are presented by the steward to the Guardians at their meetings. If approved by the Guardians, orders are given by the clerk upon the contractor appointed to supply. In the case of perishable articles such as milk, fish, fruit, etc., in respect of which a daily delivery is essential, it is impossible for the clerk's order to state the exact weekly quantity required owing to the daily variation of the number of patients maintained. In these cases the weekly order is for " fish as required," etc., and the steward either forwards a " written requisition " to the contractors or telephones the daily requirements. Although this is not a good method to adopt, the report states it cannot under the existing regulations be done away with, but the deputy clerk suggests that for the future all regular daily ordering should be in writing. It has been found that after the daily requisition has been despatched to the contractor notice has frequently been received of a convoy of patients being sent to the hospital. In such a case as this he does not see how it would be possible to obtain the additional immediate supplies required for the new patients unless the telephone be used. As a check upon this "emergency ordering" he recommends that the contractors be requested to forward to the clerk's office a duplicate of the delivery note for goods ordered by telephone, and that the delivery notes be submitted to the committee at each meeting. He is of opinion that the order " grocery as required " should be discontinued, and the steward's requisition book should contain details of the " grocery " to be ordered each week.

### LACK OF CENTRALISATION.

As to the method of receiving goods, the report states that the principal fault in the existing system is that no one person has been responsible for receiving stores delivered. One man should be held directly responsible to the steward for all matters connected with the receipt and issue of stores. All deliveries should be made at one entrance if possible, and all matters other than the actual receipt and issue of stores should be dealt with in one office.

### THE ISSUE OF STORES.

The report deals in detail with methods of issuing goods. As to stores issued to wards, it states that certain articles such as tea, sugar, oatmeal, etc., are issued direct to the wards for consumption by the patients. The quantity so issued is entered in the steward's weekly provisions consumption account, but the system is weak inasmuch as there is no proof that the quantities sent ever reach the wards. It is suggested that the responsible sister should sign a receipt for all goods received. In the majority of wards there are scales that could be used for weighing provisions received, but they should be provided in all wards.

Regarding stores issued to kitchens it is explained that the quantities sent for patients' diet are governed by the diet sheets made out by the sister in charge of each ward. As the nature of the goods issued depends upon the particular diet upon which a patient is placed by the medical officer, and the quantities constituting a diet are clearly laid down for each meal, there does not appear to be any additional check required upon the goods issued, but the same weakness again occurs as regards the receipt of the quantities. The cooks receive goods, but keep no record whatever of the quantities, and the deputy-clerk suggests that in this case also the goods delivered to the kitchens should be checked as to weights, measures, etc., and a receipt signed showing the articles and quantities received.

### STORES FOR THE STAFF.

As regards stores issued to staff officers the following observations are made: This is the most serious question of all, and the present system should be entirely abolished. Briefly, the existing method consists of sending quantities of goods to the staff quarters without any reference to a scale of rations for each officer. The quantities of goods sent are entered in the weekly provisions, receipt, and consumption account, but there is no basis for arriving at the figures so entered, and the officials at the staff quarters have no knowledge either of the quantities they ought to receive or the quantities charged in the books as being issued to them. The board will appreciate that a dishonest person could enter any figures in the book, and only issue to the staff quarters a part of the quantities charged. As it is of the utmost importance to verify that the quantities charged in the books are actually received at staff quarters, the deputy-clerk recommends that the assistant matron (home sister) be made responsible for all the staff issues, and that she be instructed to check the weight, measure, etc., of the goods and sign a receipt for them.

### WEEKLY EXAMINATION OF ACCOUNTS AND BOOKS.

The following is Mr. Owen's reference to examination of books:

" In the event of the foregoing suggestions being acceptable to the Guardians, I recommend that in future all delivery notes be sent to the clerk's office weekly, in order that the quantities signed for may be examined with quantities charged upon the invoices. The various books should then be checked with the invoices to see that the new stock is correctly entered.

" Receipts for stock issued should also accompany the books, and the quantities signed for checked with the amounts shown as issued.

" I also recommend that a book to be known as the 'Report upon Examination of Day Book, etc.,' be pre-

**HOW NOT TO RUN A WAR HOSPITAL—(*cont.*)**

sented to the Finance Committee at their ordinary meetings.

"This book should contain the written result of the weekly examination of the steward's day-book, invoices, delivery notes, receipts for stores issued, provision receipt and consumption accounts, etc., and attention should be called therein to any inaccuracy in the matters under consideration."

SUGGESTED RULES.

Generally, the report makes the subjoined suggested rules for consideration:

"*Hours for Issuing Stores.*—Stores to be issued between the hours of 8 A.M. to 11 A.M., and 3 P.M. to 5 P.M., except in cases of emergency. During the remainder of the day the stores are to be kept locked.

"*Persons Entering the Stores.*—No person is to enter the stores at any time in the absence therefrom of the steward or assistant steward.

"*Removal of Parcels, etc.*—No male officer or servant, or member of the female domestic staff, or inmate of the workhouse will be allowed to take, or cause to be taken from the premises, any parcel, box, bag, package, or property belonging to the Guardians, without the written consent of the officer commanding, steward, or matron.

"*Delivery of Day Book, etc., to Clerk's Office.*—The steward shall send, complete and entered up to date, all or such of his books, etc., as may be required, to the clerk's office for examination at 10 A.M. every Friday, and at any other time if required by the clerk or deputy-clerk so to do.

"In reference to stocktaking, the procedure observed at present is for the stocktaker to take stock at the end of each quarter. I consider it would be a good plan if a 'surprise stocktaking' took place once *during* each quarter, upon a date convenient to the stocktaker, to be privately selected by the Chairman of the Board."

